# Development of a Novel Electrochemical Sensor Based on Gold Nanoparticle-Modified Carbon-Paste Electrode for the Detection of Congo Red Dye

**DOI:** 10.3390/molecules28010019

**Published:** 2022-12-20

**Authors:** Aisha Ganash, Sahar Alshammari, Entesar Ganash

**Affiliations:** 1 Chemistry Department, Faculty of Science, King Abdulaziz University, Jeddah 23714, Saudi Arabia; 2 Physics Department, Faculty of Science, King Abdulaziz University, Jeddah 23714, Saudi Arabia

**Keywords:** Congo red dye, carbon-paste electrode, gold nanoparticles, electrodeposition, electrochemical sensor

## Abstract

In this study, gold nanoparticles (AuNPs) were electrodeposited on samples of a carbon-paste electrode (CPE) with different thicknesses. The prepared AuNPs were characterized using different analysis techniques, such as FTIR, UV–Vis, SEM, EDX, TEM images, and XRD analysis. The fabricated modified electrode AuNPs/CPE was used for the sensitive detection of Congo red (CR) dye. Electrochemical sensing was conducted using square-wave voltammetry (SWV) in a 0.1 M acetate buffer solution at pH 6.5. The proposed sensor exhibited high efficiency for the electrochemical determination of CR dye with high selectivity and sensitivity and a low detection limit of 0.07 μM in the concentration range of 1–30 μM and 0.7 μM in the concentration range of 50–200 μM. The practical application of the AuNPs/CPE was verified by detecting CR dye in various real samples involving jelly, candy, wastewater, and tap water. The calculated recoveries (88–106%) were within the acceptable range.

## 1. Introduction

A large increase in human presence and human activities has led to an increase in water pollution and loss of water quality. The detection and removal of unwanted and hazardous materials has been considered a major challenge in recent years. Many efforts have been adopted to detect and remove toxic substances to reduce water pollution. Moreover, biological and environmental issues have been produced by textile industry effluents. Other products, such as cosmetics, paint, lather, foodstuff, and paper, also involve organic dyes [1,2,3,4]. Different types of dyes, such as thiazol, diazo, azo, and quinine imine, have been used in the textile industry and disposed of as effluents. Commonly called Congo red (CR), this anionic azo dye [1-naphthalenesulfonic acid, 3,3′-(4,4′-biphenylene bis(azo)) bis (4-amino-) disodium salt] has two azo groups and a complex structure that is responsible for the intense color that resists fading under washing, soap and light exposure [5]. In general, CR has been historically used as a clothing dye, as an indicator, in cosmetic synthesis, and food. It is unsafe and toxic, and it can lead to cancer, cause damage to living organisms, cause an allergic reaction in the eye, and lead to kidney, liver, and bladder dysfunction [6,7]. Different techniques have been used to detect and analyze CR, such as fluorescent sensors [8], high-performance liquid chromatography [9,10], UV–Vis spectrophotometry [11,12], and electrochemical measurements [13]. Various methods have been used to analyze trace dye concentrations, such as capillary electrophoresis, separation methods, and photometric measurements. However, some of these techniques are not convenient for routine measurement owing to their complications, time consumption, and poor selectivity and sensitivity. In this context, it is worth mentioning that the electrochemical technique is simple, rapid, has high sensitivity and selectivity, and low cost.

Nanoparticles with small sizes and high surface areas reveal unique properties when compared with bulk materials, and these properties make them useful for different applications [14,15,16,17,18,19,20]. Gold nanoparticles (AuNPs) have attracted much attention in recent years for different applications due to their optoelectronic and catalytic activity. In addition, AuNPs can be used to improve electrical conductivity.

**Scheme 1:**
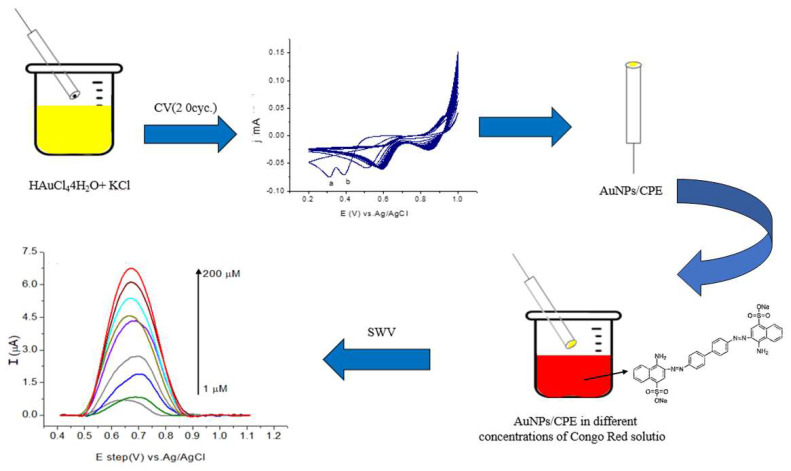
Process steps of the electrochemical detection of CR.

A novel AuNP/CPE electrochemical was constructed by direct electrodeposition of AuNPs on a carbon-paste electrode (CPE) surface using the cyclic voltammetry (CV) technique. The modified electrode was used as a simple and sensitive sensor for the detection of CR dye in various real samples (Scheme 1).

## 2. Results and Discussion

### 2.1. Characterization

#### 2.1.1. Fourier Transform Infrared (FTIR) and Ultraviolet-Visible (UV–Vis) Spectroscopy

**Figure 1:**
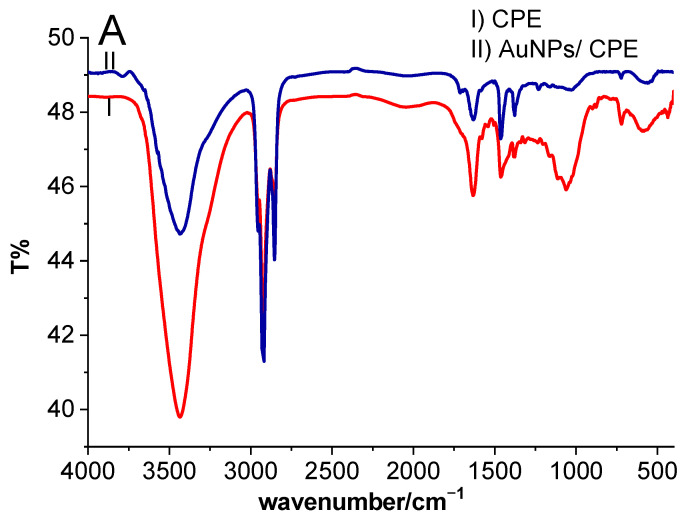

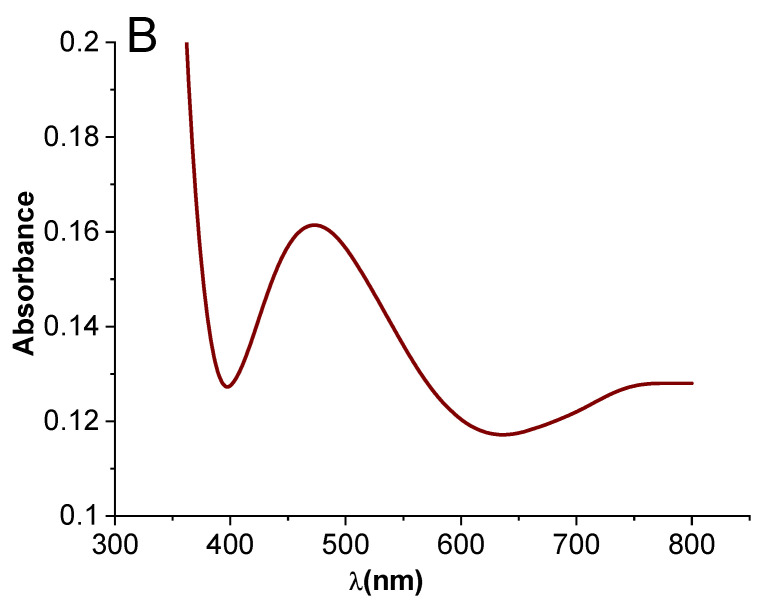
(**A**) FTIR spectra of the (I) CPE and (II) AuNPs/CPE. (**B**) UV–vis absorbance of AuNPs.

FTIR analysis was used to identify the different functional groups involved in the compounds. FTIR was carried out by crushing all samples with KBr pellets. The analysis was performed in the region of 4000–400 cm^−1^ using a Perkin Elmer spectrometer (Waltham, MA, USA). Figure 1A compares the FTIR absorption spectra of the CPE before (Figure 1 A(I)) and after (Figure 1 A(II)) the electrodeposition of AuNPs/CPE. As shown, different bands appeared at different wavenumbers. In (Figure 1 A(II)), a strong and broad band appeared at 3452 cm^−1^, denoting the O-H stretching vibration [3]. This absorbance band became less boarding and shifted to 3448 cm^−1^ after the addition of AuNPs. Additionally, two sharp bands at 2926 cm^−1^ and 2849 cm^−1^ indicate asymmetric and symmetric C-H alkane stretching vibrations (CH_2_ groups), respectively [1], while after AuNP addition, both bands shifted to increase wavenumber to 2927 and 2857 cm^−1^, correspondingly. The absorbance band, which appeared at 1645 cm^−1^ before adding AuNPs moved to 1637 cm^−1^ with the existence of AuNPs. This band indicates the C=C stretching vibration [3]. The band at 1447 cm^−1^ is attributed to the C-C alkane vibration [4] and shifted by about 10 cm^−1^ when AuNPs were added (1457 cm^−1^). This reflects the vibrations of alkanes (carbohydrates) ending in CH_2_ and indicates C–C ring vibrations for aromatic groups [4]. Moreover, the band at 1051 cm^−1^ indicates the stretching vibration of C-O [1], while after depositing the AuNPs the band centered at 1028 cm^−1^ which means there is a big change in wavenumbers [2]. Hence, it is clear after the addition of AuNPs, the intensities of all bands decreased except two bands at 2926 cm^−1^ and 2849 cm^−1^, which increased. Additionally, we found the bands became narrow, and there is a shift in the wavenumber position due to the presence of AuNPs. Furthermore, a new peak appeared at 1380 cm^−1^. This is attributed to the reaction between the CPE and AuNPs that takes place [3] and indicates O-H bending vibration [1].

UV–Vis spectroscopy is a qualitative and nondestructive technique that measures the optical properties such as the absorption or reflectance of materials in the UV–Vis spectral region. This technique measures electronic transitions from the ground state to the excited state of a molecule after absorption of electromagnetic waves in the ultraviolet and visible regions. Thus, π-electrons or nonbonding electrons (n-electrons) in the molecules can absorb the energy in the form of UV–Vis waves to excite these electrons to higher antibonding molecular orbitals. The more easily excited the electrons are, the longer the wavelength of light that can be absorbed. UV–Vis spectroscopy is commonly used to monitor the formation of nanoparticles [21]. Metal nanoparticles are characterized by the surface plasmon resonance (SPR) band. This peak is a result of the interaction of the incident light at a specific wavelength in the resonance process and the collective oscillation of electrons in the conduction band of the NPs. As described, the SPR spectrum depends on the size and shape of the nanoparticles; therefore, the size can be estimated by knowing the position of this peak. As the size decreases the SPR shifts toward short wavelength [22,23]. As shown in Figure 1B the SPR is seen at around 473 nm, which indicates the existence of some AuNPs with spherical shapes and a size of around less than 10 nm.

#### 2.1.2. Scanning Electron Microscopy (SEM) and Energy-Dispersive X-ray Spectroscopy (EDX)

**Figure 2:**
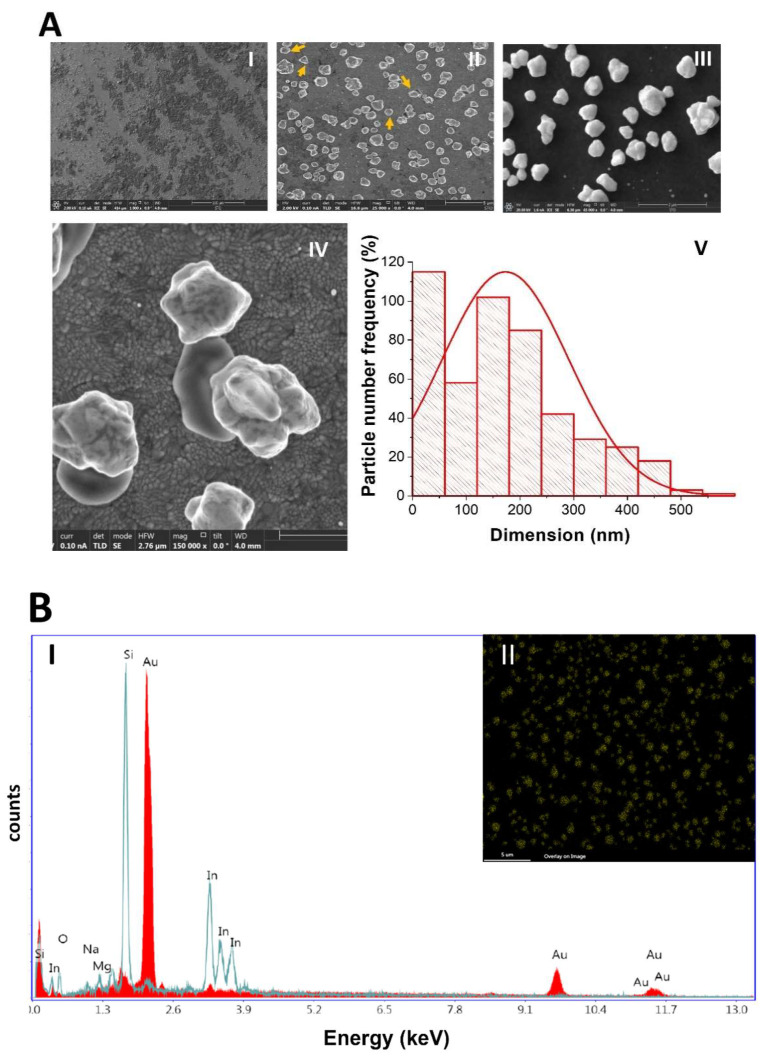
(**A**) SEM images at different magnifications of (I) 1000×, (II) 25,000×, (III) 65,000× and (IV) 150,000×. (V) The size distribution of AuNP images from SEM. (**B**) (I) EDX spectrum of AuNPs and (II) SEM/EDX mapping distribution of Au.

Scanning electron microscopy (SEM) analysis is used to determine the morphology of the surface of the synthesized gold nanoparticles. It was applied to scan the resulting sample with an energy beam at 20 and 2 keV. The images were taken at different magnifications, as displayed in Figure 2A(I–V). The SEM photographs show that the fabricated gold nanoparticles have surfaces with several faceted shapes. These nanoparticles do not have an exact spherical shape. Some of them appear semispherical, spherical, hexagonal, pentagonal, and triangular (Figure 2A(II)). These different shapes are due to how they sit on the glass substrate. As shown from the particle size distribution (Figure 2A(V)), the size range varies from 15 nm to 542 nm, with an average size of approximately 157 nm.

The EDX spectrum of the fabricated nanomaterials is shown in Figure 2B(I). That spectrum clearly shows the strong signal that indicates the existence of gold as the primary element. The results reveal that the composition was approximately 99.46 ± 1.92 wt.% of gold. The distribution of gold is shown in Figure 2B(II) using the EDX-based SEM image with a 5 μm scale. This result clearly proves the effective formation of gold nanoparticles. The presence of silicon, indium, and oxygen elements is expected due to the use of ITO-coated glass as a substrate for the fabricated materials. Moreover, the percentages of other elements, such as Na and Mg, are very low. These elements might be produced in the sample; however, these are beyond our objective in this work.

#### 2.1.3. Transmission Electron Microscopy (TEM)

**Figure 3:**
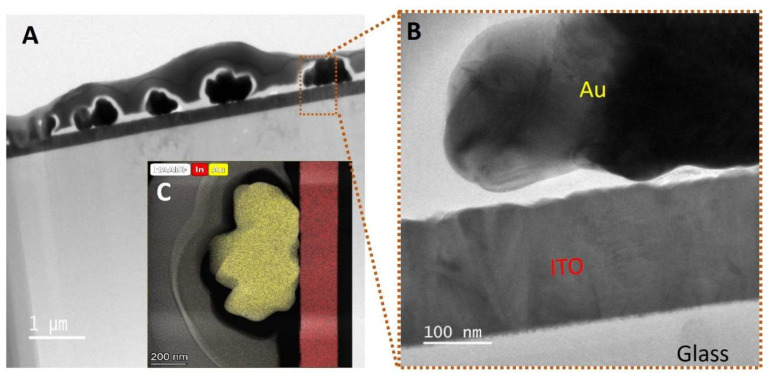
(**A**) TEM image of AuNPs, (**B**) magnified image of AuNPs and (**C**) TEM cross-section HAADF of AuNPs.

In addition, TEM analysis was applied. Figure 3A shows TEM cross-section bright field images of the nanogold particles created on the ITO substrate. These nanogold particles overlapped and aggregated highly with each other, as shown by the dense black area in Figure 3B. The high-angle annular dark field (HAADF) of the TEM cross-section image is also displayed in Figure 3C the yellow color indicates nanogold particles, while the red color indicates indium (In) metal.

#### 2.1.4. X-ray Diffraction Spectroscopy (XRD)

**Figure 4:**
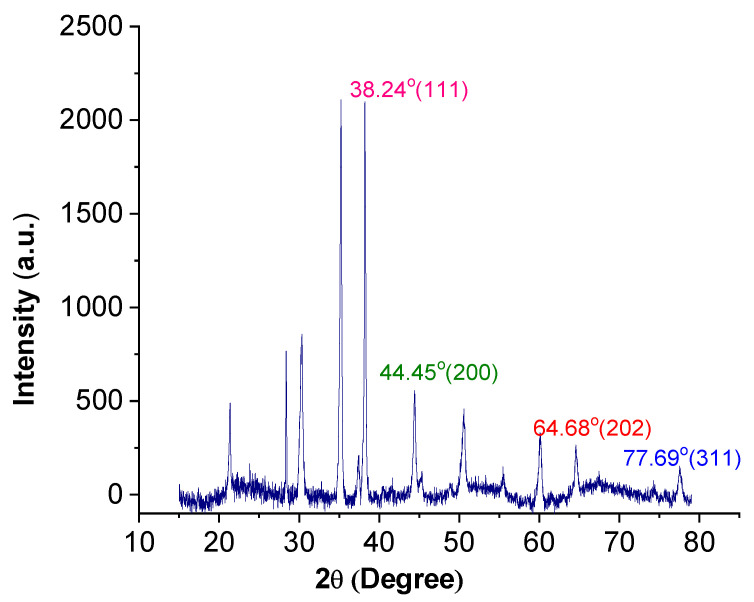
XRD spectrum of AuNPs.

XRD technique was used to examine the crystallinity of AuNPs deposited on ITO conductive glass (1.5 cm × 1.5 cm). The XRD spectrum is shown in Figure 4. It is clear that AuNPs are represented in four definite sharp peaks at two-theta values of 38.24°, 44.45°, 64.68°, and 77.69° corresponding to different crystal orientations of (111), (200), (220), and (311), respectively. As reported, the intense sharp diffraction peak at 38.24° indicates that the orientation of Au^0^ growth in the (111) direction is preferred. This result denotes that the size of the formed solid molecule with a repeating 3D pattern is uniform [24]. The resulting XRD graph represents a classic pattern for pure AuNPs [25,26,27,28] upon comparison of the XRD pattern with the JCPDS database file number 00-004-0784.

### 2.2. Electrochemical Activity Investigation

**Figure 5:**
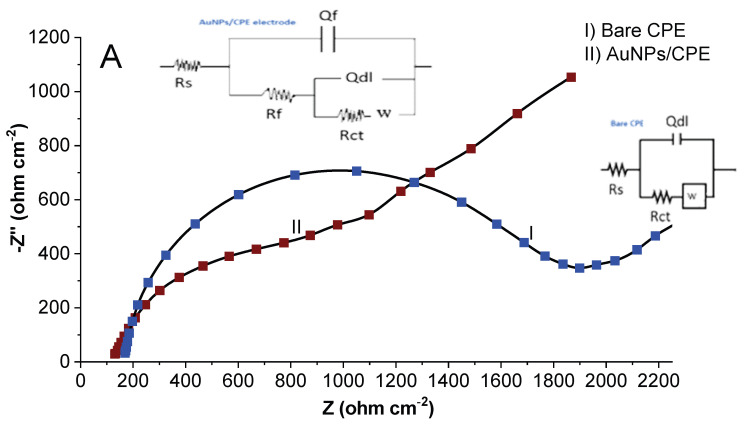

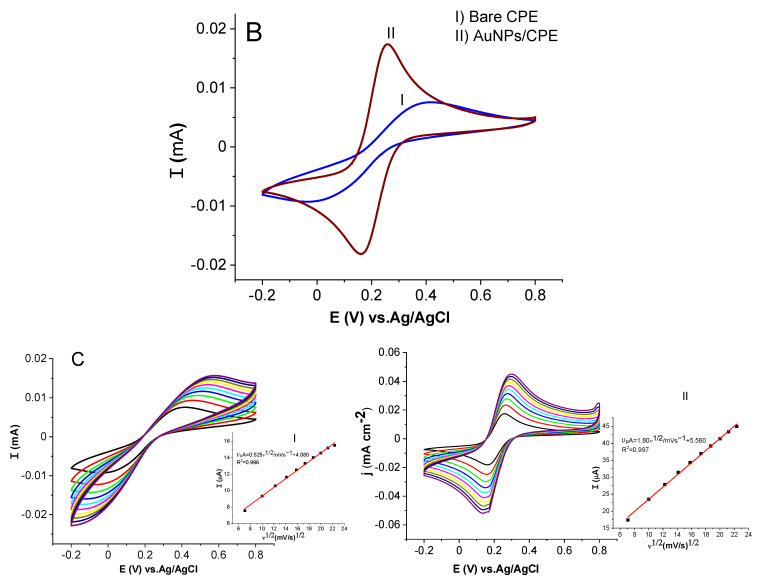
(**A**) Nyquist graphs of EIS measurements with the fitted equivalent circuits (insert figures), (**B**) CV graphs at 50 mV/s, and (**C**) CV in 2 mM [Fe(CN)6]^3−/4−^ and 0.1 M KCl solution at various scan rates of the (I) bare CPE and (II) AuNPs/CPE. Inset: Peak currents as a function of the square root of the potential.

The electrochemical behavior of the bare CPE- and AuNP/CPE-modified electrodes was studied in 2 mM K_3_[Fe(CN)_6_] in 0.1 M KCl solution using CV and EIS measurements. Figure 5A indicates that all electrodes show depressed semicircles in the high-frequency region, and the diffusion of [Fe(CN)_6_]^3−^ ions toward the electrode was clearest as a diffusion tail in the low-frequency region. The best-fitting equivalent circuits represent the formed electrical circuit at the electrolyte|electrode interface inserted in Figure 5A. The equivalent circuits are summarized as R_s_ (Q_dl_ (R_ct_ Z_w_)) for the bare CPE and as R_s_ (Q_f_ (R_f_ (Q_dl_ (R_ct_ Z_w_)))) for the AuNP/CPE-modified electrode, where R_s_ is the solution resistance, R_ct_ is the charge transfer resistance, R_f_ is the film resistance, Z_w_ is the Warburg resistance (mass transfer resistance), and Q_f_ and Q_dl_ are constant face elements for the film and the electrical double layer, respectively. The modification of the electrode by AuNPs causes a significant drop in the R_ct_ (896 Ω) compared with the R_ct_ of the bare CPE (1613 Ω), which indicates rapid electron transfer on the AuNP/CPE surface.

The CV measurements were used to support the EIS results for the bare CPE and AuNP/CPE-modified electrode. As seen in Figure 5B, the modified electrode exhibits a redox current of 0.017 mA/cm^2^ in the anodic direction and 0.018 mA/cm^2^ in the cathodic direction, which is higher than that of the bare CPE. A similar result has been observed and reported [19]. These results are expected due to the high surface area, high activity, and conductivity of the AuNPs [29].

The effective surface area of the bare CPE and AuNP/CPE-modified electrode was estimated using different scan rates (50–500 mV/s) of CV measurements, as shown in Figure 5C. The Randles–Sevcik equation (Equation (1)) was used, and the effective area was calculated from the slope as 4.5 × 10^−3^ cm^2^ for the bare CPE and 13.5 × 10^−3^ cm^2^ for the AuNP/CPE.

(1) $$i_{p}=2.69\times 10^{5}An^{3/2}D^{1/2}Cv^{1/2}$$

in this equation, *i_p_* is the current, *ν* is the scan rate, *C* is the concentration of K_3_[Fe(CN)_6_] in mol/cm^3^, *n* = 1, and *D* = 7.6 × 10^−6^ cm^2^/s. The constant term 2.687 × 10^5^ has the unit (C·mol^−1^/V^1/2^), and *A* is the effective surface area. This result demonstrates that the modified electrode acquires a high surface area compared with the bare electrode; thus, the high electrochemical activity of the modified electrode is expected.

### 2.3. Optimization of Investigational Parameters

#### 2.3.1. Influence of Changing pH and the Supporting Electrolyte

**Figure 6:**
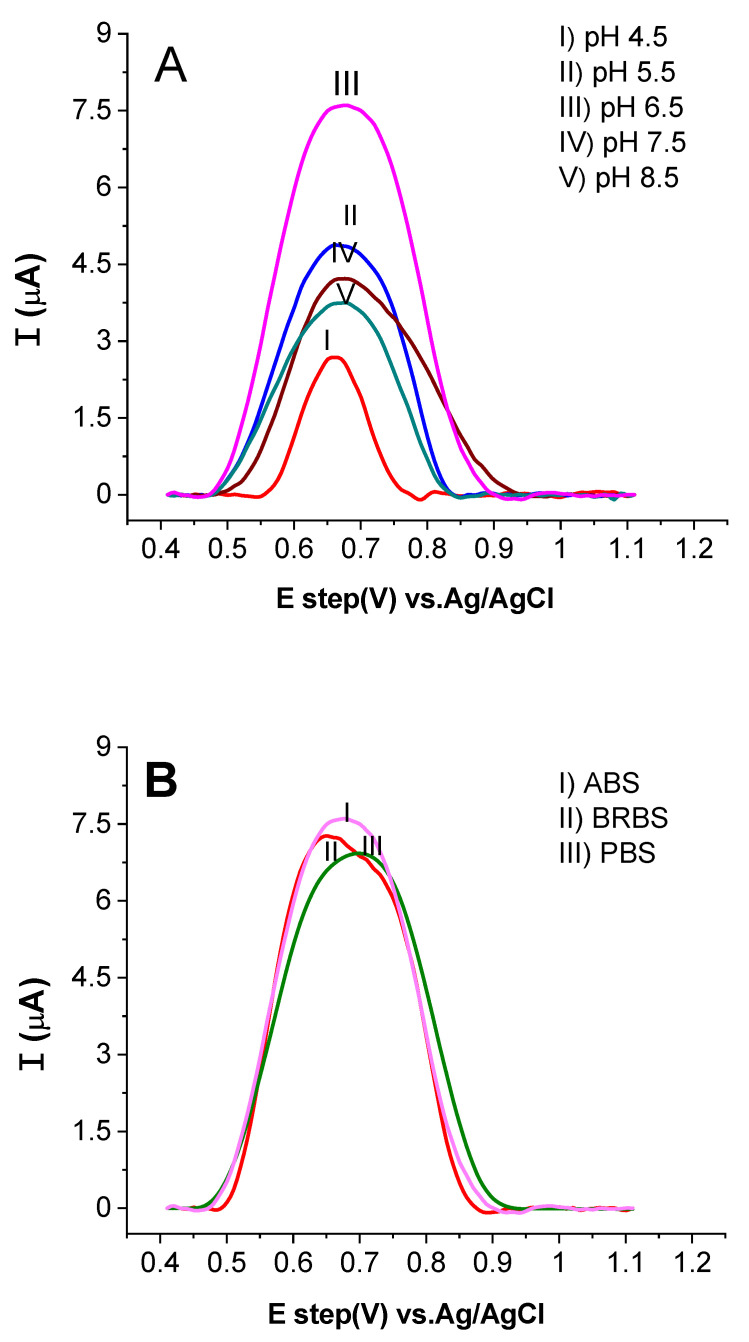
SWV measurement of 250 μM CR (**A**) in 0.1 M ABS at different pH values and (**B**) in different buffer solutions at pH 6.5.

The effect of the change of pH value of 250 μM CR in 0.1 M acetate buffer solution on the current response was investigated in the pH range of 4.5 to 8.5, as shown in Figure 6A. As seen from the figure, the current response increased from an acidic medium (4.5) to a mostly natural medium (6.5), after which a further increase in pH value led to a decrease in the current response, and the lowest current response was recorded at pH 8.5; therefore, pH 6.5 was selected for future measurement. As reported CR consider an anionic molecule at alkaline pH due to the dissociation of the sulfonate group, while in an acidic medium CR is a cationic molecule [30]. In the highly acidic medium, excess H^+^ ions are present in the solution which can accumulate around negatively charged AuNPs. The cationic CR is weakly attached to the negative surface of the AuNPs and competes with H^+^ for available sites at acidic pH, a repulsion process accrues between the cationic CR and the surrounding H^+^ ions [31]. However, the increase in pH to 6.5 leads to a decrease in the H^+^ ions and an increase in the unprotonated form of CR molecules around the electrode, which enhances the oxidation of the current signal [32]. The depletion of the oxidation current signal in the high basic medium may be ascribed to the repulsion between the negatively charged AuNPs and both OH^−^ ions and the anionic form of CR [33].

Additionally, 0.1 M of different buffer solutions, such as phosphate buffer solution (PBS), acetate buffer solution (ABS), and Britton–Robinson buffer solution (BRBS), were tested at pH 6.5, as depicted in Figure 6B. The maximum oxidation current response was observed in 0.1 M ABS. Thus, to maximize the signal of the oxidation current, the 0.1 M ABS at pH 6.5 was adopted for further measurements.

#### 2.3.2. Effect of AuNP Thickness Layer

**Figure 7:**
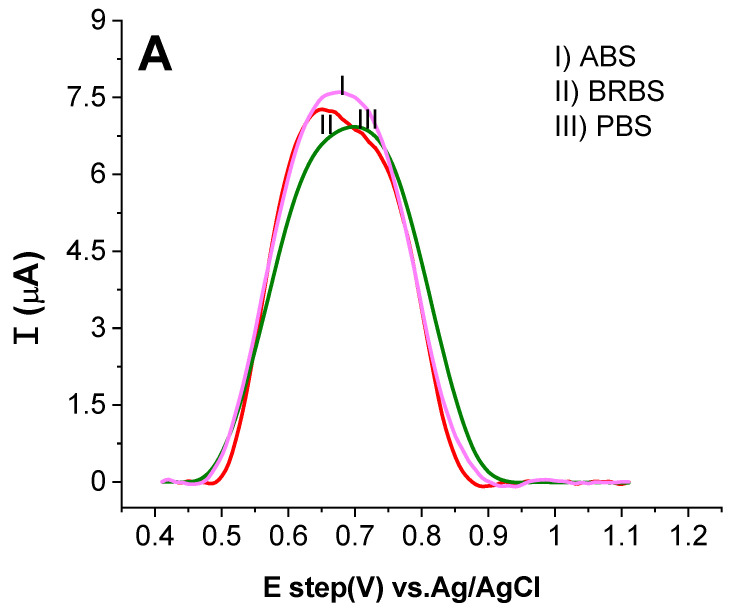

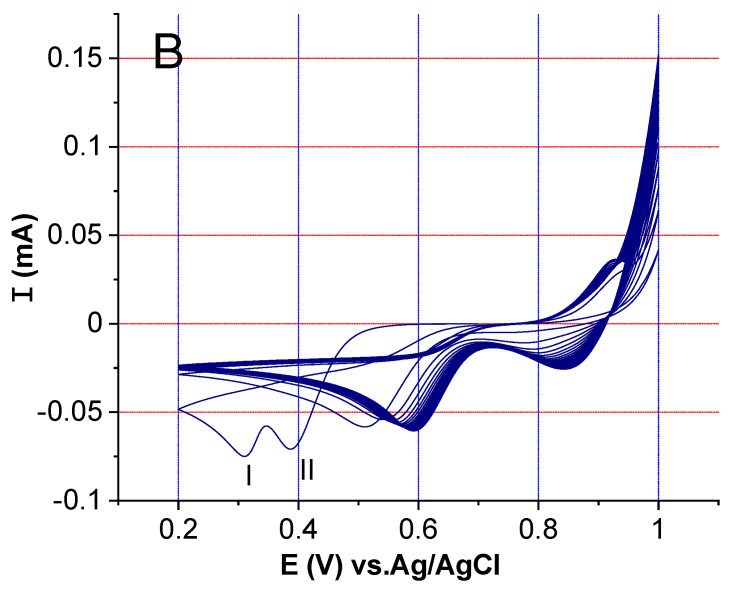
(**A**) Effect of the AuNP electrodeposition cycle number on SWV curves of 250 μM CR in 0.1 M ABS at pH 6.5 and (**B**) CV of the electrodeposition of AuNPs.

For the preparation of AuNPs as a thin and adherence layer on the top surface of the electrode, the clean and polished CPE was directly immersed in a solution of 2 mM HAuCl_4_·4H_2_O in 0.1 M KCl at room temperature, After that the CV sweep was carried out in the potential range of 0.2 to 1.0 V with desired cyclic number at a scan rate of 50 mV/s. Various numbers (4, 8, 12, 16, 20) of CV cycles in the potential range of 0.2–1.0 V at 50 mV/s were performed; the electrode was allowed to dry and was then immersed in 250 μM CR in 0.1 M ABS at pH 6.5, and the SWV response is depicted in Figure 7A. The figure shows that the high-thickness layer demonstrates a higher current response. This outcome may be due to an increase in the active surface area of the nanoparticles on the electrode surface. The concentration of active AuNPs on the electrode surface can be calculated from the reduction peak using Equation (2) [34].

(2) $$I_{P}=\frac{n^{2}F^{2}A\Gamma v}{4RT}$$

where *I_p_* is the reduction current (A), *A* is the area of the electrode surface (0.196 cm^2^), *ν* is the scan rate (0.05 V/s), *n* is the reduction electron number (3e), *R* = 8.3145 J/k mol, *T* = 298 K, and *F* = 96,485 C/mol. The calculated Γ values are 6.16 × 10^−10^, 6.52 × 10^−10^, 6.88 × 10^−10^, and 7.00 × 10^−10^ cm^2^/mol for 4, 8, 12, 16, and 20 cycles, respectively. These results demonstrate that the increase in the number of cycles provided more layer thickness.

The CV graph of the electrodeposition is presented in Figure 7B. The first cycle indicates two reductive peaks I and II at approximately 0.313 V and 0.390 V due to the reduction of Au(III) to Au(0) as nanoparticles and the reduction of protons [35,36,37]. For the backward direction, the scan intersects at 0.451 V. Beyond this point, the current increased, and this result is a strong indication that the growth of previously formed Au nanoparticles is more likely than the formation of new particles on the electrode surface [38,39]. Notably, the shift in the cathodic peak during the sequence cycle emphasizes that the electrodeposition process appeared preferentially on the first-formed layer of AuNPs [40]. Referring to previous work, the potential intersection point is related to the polishing of the electrode surface since this intersection point is not observed when the surface is not adequately polished [41]. Moreover, the sequence cycles show a lower cathodic peak current; this outcome is mostly a result of AuCl^−^_4_ depletion in the diffusion layer [40], and a similar picture has been obtained [27]. At the end of the measurements, the Au color was observed.

#### 2.3.3. Influence of Accumulation Time

**Figure 8:**
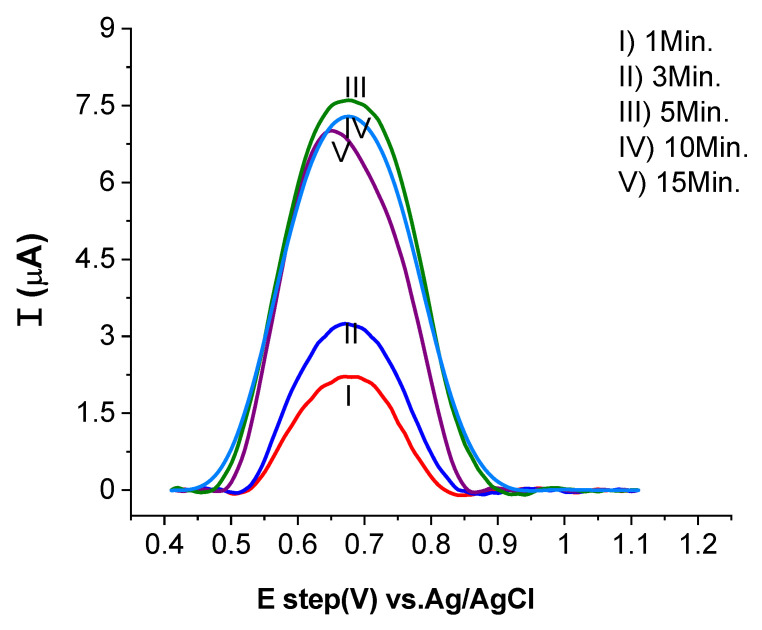
Effect of the accumulation time on SWV curves of 250 μM CR in 0.1 M ABS at pH 6.5.

The most suitable effective time to analyze CR dye was evaluated in the range of one minute to 15 min (Figure 8). It is clear that the current signal increased for the first five minutes, and soon after that, the current signal decreased again. This result is ascribed to the saturation of the AuNP/CPE-modified electrode with CR molecules and the competition phenomena among dye molecules to adsorb on the electrode surface, as well as to the fouling of the Au/CPE electrode [42].

#### 2.3.4. Study of the Effect of Scan Rate Change

**Figure 9:**
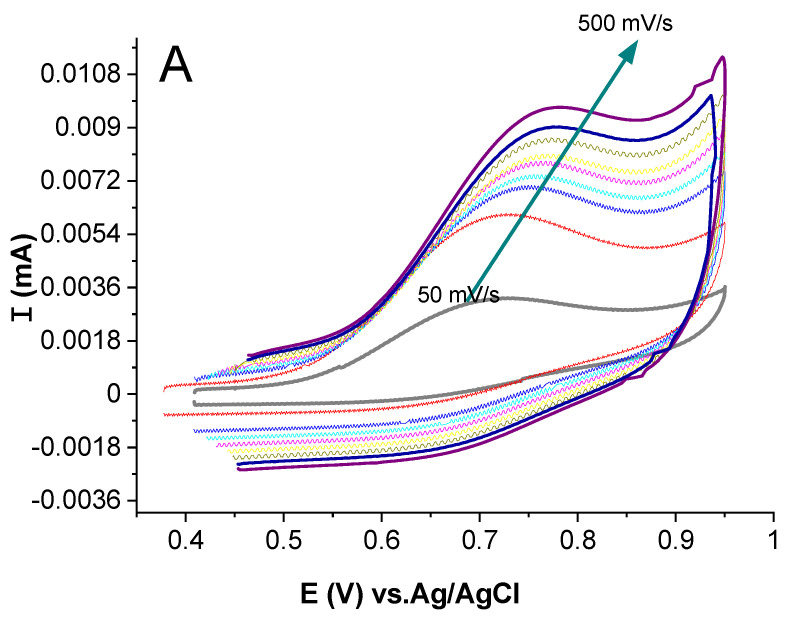

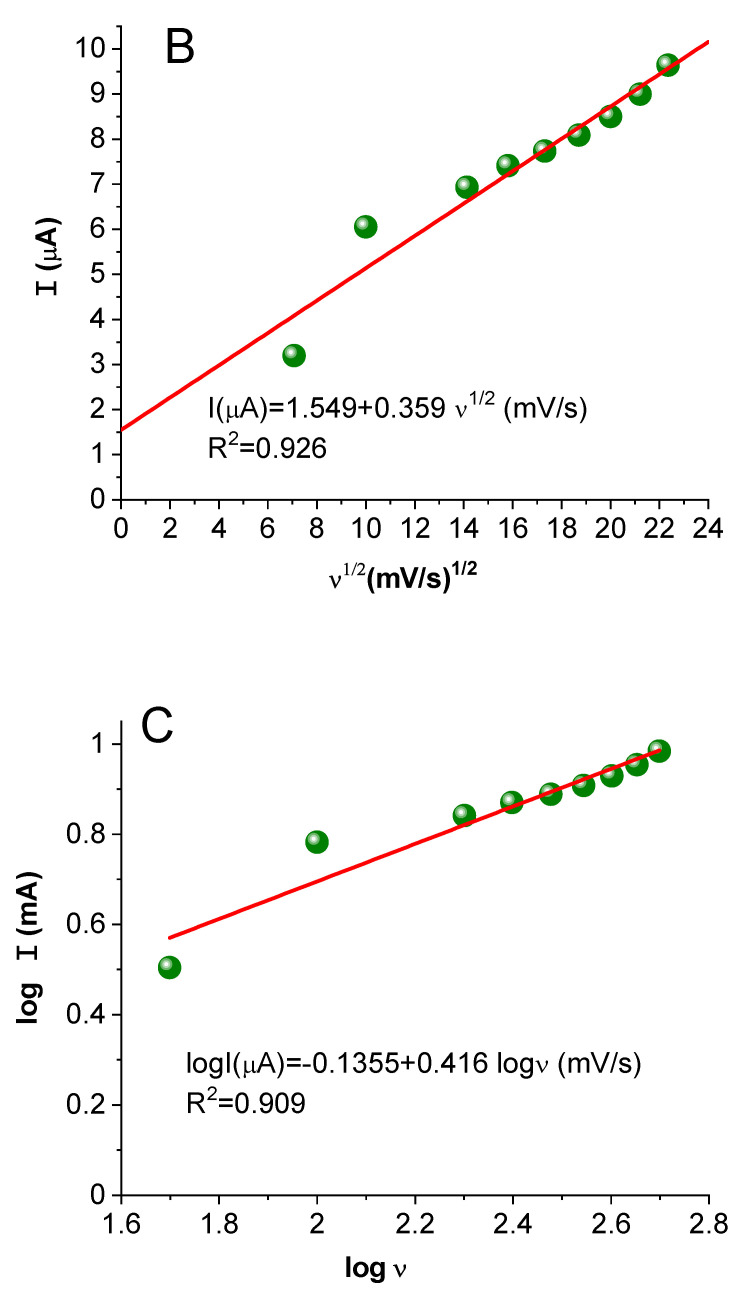

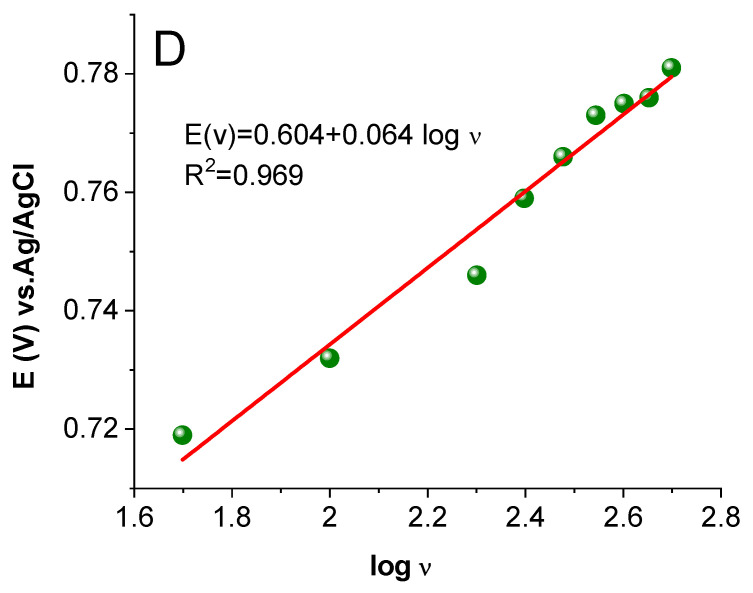
(**A**) Effect of change in scan rate on the CV in the presence of 250 μM CR in 0.1 M ABS at pH 6.5, (**B**) plot of current vs. ν^1/2^, (**C**) plot of log I vs. log ν and (**D**) plot of E vs. log ν.

To explore the electrochemical mechanism for the oxidation of CR on the AuNP/CPE electrode surface, CV of 250 μM CR in 0.1 M ABS at pH 6.5 was performed at different scan rates in the range of 150–500 mV/s, as represented in Figure 9A. The figure reveals one irreversible anodic peak and the absence of a cathodic peak; the peak current increases, and the peak potential shifts to a more positive value with the regular increase in scan rate. This shift emphasizes the irreversibility of the oxidation process [43].

The linear relationship between the oxidation current and the square root of the scan rate was produced [I(μA) = 1.549 + 0.359 ν^1/2^(mV/s)^1/2^], as shown in Figure 9B. This situation suggests that the diffusion process demands the oxidation of CR [44,45]. Furthermore, the plot of log I versus log ν (Figure 9C) yields a slope of 0.416 (μA/mVs^−1^) with the relationship of log I (μA) = −0.1355 + 0.416log ν (mV/s). It was stated that the linear relationship creates by plotting log I versus log ν with a slope of 0.5 for the pure diffusion control process and a slope of 1.0 for pure adsorption control process, the slope value between 0.5 and 1.0 correspond to process under diffusional/adsorption control [46]. In this study, the slope is 0.416 which proves that the oxidation of Congo red is under partial diffusion control. The plot of anodic peak potential (Ep) versus log ν (Equation (3) and Figure 9D) [i.e., E(V) = 0.604 + 0.064 log ν (mV/s)] implies a linear relationship with a slope of 0.064 V. The number of electrons involved in the rate-determining step of the CR oxidation process was estimated from the Tafel slope (b) using Equation 3 and b = 2*slope = 2 × (0.064) = 0.128 Vdec^−1^ (The Tafel slope close to 120 mVdec^−1^, since RT/F = 59.2 or ≈60 mV at 25 °C), which confirms that as the process proceeds through the transfer of one electron in the rate-determining step [47,48,49]. α = 0.5 was calculated from Equation (4) [50].

(3) $$E_{p}=\frac{b}{2}\;log\nu +constant$$

(4) $$b=\frac{2.303RT}{\left(1-\alpha \right)nF}$$

in this equation, *n* is the number of electrons transferred; *F*, *R*, and *T* have their usual meaning; and α is the heterogeneous rate constant of electron transfer between the electrode and the deposited layer, which was calculated from the intercept [13].

#### 2.3.5. Electrochemical Sensing of CR on the AuNPs/CPE

**Figure 10:**
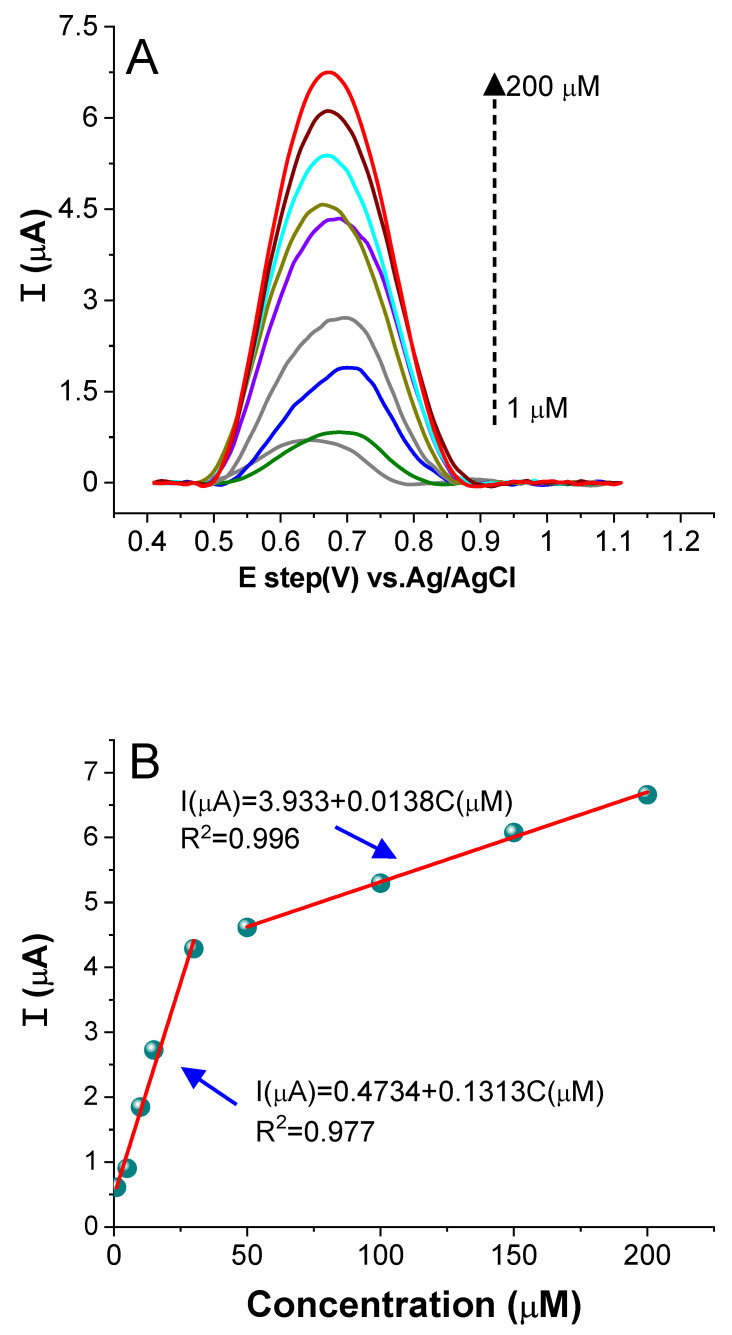
Effect of change of CR concentration on (**A**) SWV and (**B**) oxidation current signal in 0.1 M ABS solution at pH 6.5.

A series of concentrations of CR in the range of 1.00 to 200 μM in 0.1 M ABS at pH 6.5 was used under optimum conditions, as represented in Figure 10A, to expose the catalytic effect of the AuNP/CPE electrode. Please note that the current response increased directly with concentration, and the regression equation I(μA) = 0.4734 + 0.1313C(μM) (R^2^ = 0.977) in the range of 1.0–30 μM and I(μA) = 0.4734 + 0.1313C(μM) (R^2^ = 0.996) in the range of 50–200 μM was achieved.

The limit of detection (LOD) and limit of quantitation (LOQ) were estimated as 3*SE/slope and 10*SE/slope, respectively [51], where SE is the standard error from three blank measurements and the slope of the calibration curve (Figure 10B). The modified sensor possesses an excellent LOD = 0.07 in the concentration range of 1.0–30 µM and 0.7 in the concentration range of 50–200 µM with LOQs of 0.25 and 2.4. A comparison of the Au/CPE with the previous sensor is tabulated in Table 1.

### 2.4. Stability, Reproducibility, and Interference Study

**Figure 11:**
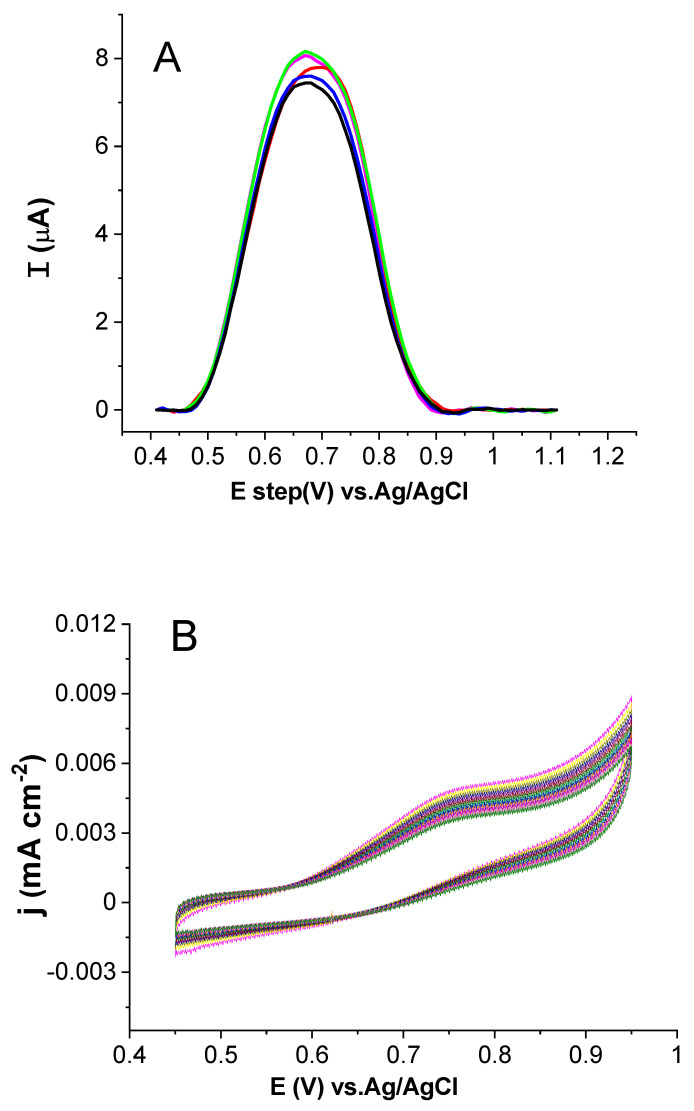

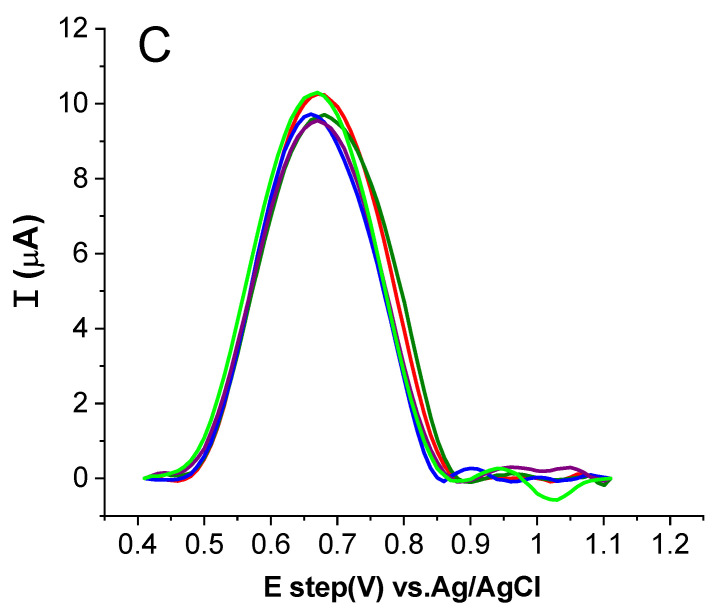
(**A**) A series of five SWV measurements, (**B**) 20 sequences of CV measurements from −0.45 to 0.95 V at 100 mV/s, and (**C**) two weeks of SWV measurements of the AuNPs/CPE in 250 μM CR in 0.1 M ABS at pH 6.5.

The crucial factors for electrochemical sensors are their stability, reducibility, and selectivity. Accordingly, these factors for the fabricated AuNP/CPE were examined in 250 μM CR in 0.1 M ABS at pH 6.5. For the reproducibility study, five electrodes were prepared and tested; the RSD was 3.920%, thus no appreciable change was detected (Figure 11A). The stability study was performed using two methods: the first method (Figure 11B) measured 20 continuous cycles using the CV technique (RSD is 9.604%) and the second method (Figure 11C) measured the SWV of the same electrode for two weeks. For the second method, a calculated RSD of 3.908% was obtained. As described elsewhere, an RSD value of less than 10% is acceptable [53,54,55].

A foreign substance can affect and interfere with the CR current signal measurement. The selectivity of the AuNP/CPE-modified electrode was assayed in the presence of 250 μM CR in 0.1 M ABS at pH 6.5 with 0, 50, 100 200, 300, 400 and 500 μM NH_4_^+^ (RSD = 1.092), Na^+^ (RSD = 1.66), K^+^ (RSD = 0.79), Zn^2+^ (RSD = 1.14), Cd^2+^ (RSD = 0.96), methyl orange (RSD = 2.22) and tartrazine (RSD = 1.12). These results indicate that the tested species did not affect or interfere with the signal in the original substance. Thus, the proposed electrochemical sensor demonstrates significant and high selectivity.

### 2.5. Practical Application of CR Recovery in Real Samples

The applicability analysis of the AuNPs/CPE in different samples, including jelly, wastewater, soil, tap water, and candy, was analyzed at room temperature. The measurement was run in one milliliter of the real sample diluted with 0.1 M ABS at pH 6.5. The recovery percentage was calculated using Equation (5):

(5) $$Recovery=\frac{C_{found}}{C_{added}}\times 100$$

where *C_found_* is the found analyte concentration detected in the spiked sample and *C_added_* is the added analyte concentration. In the spike/recovery system, a definite amount of analyte is added (spiked) into the real test sample matrix and its response is measured (recovered) in the assay by contrast to an identical spike in the standard diluent, i.e., the dependable detection was assessed by detection accuracy (recovery of insignificant gravimetric concentrations). As described a recovery more than 100% suggests that the measured values for a matrix were higher than the nominal value of the spike, and recovery less than 100% indicated that the measured values for a matrix were lower than the insignificant value of the spike [56]. However, a spike recovery value of greater than 100% means that the analytical method gives a positive error. As reported, the recovery in the range of 80–120% is appropriate and does not require matrix corrections. This range depends on the (i) nature of the samples, (ii) extraction procedure, and (iii) concentration levels of the elements [57].

The recovery was in the range of 88% to 106%, as summarized in Table 2. This result confirms that the Au/CPE is applicable to use in a real environment.

### 2.6. Mechanism

**Scheme 2:**
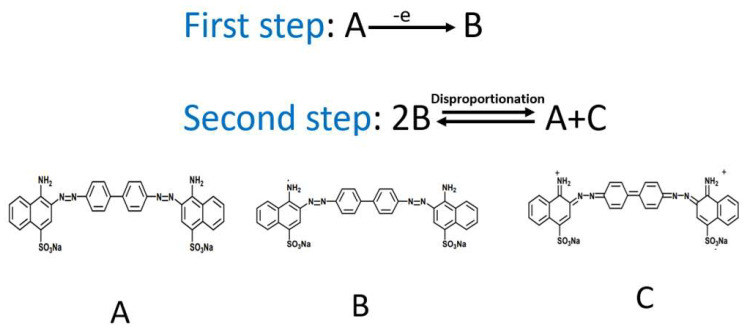
Reaction mechanism of the oxidation of CR.

It has been reported that CR exhibited a disproportionation process [58,59]. The electrochemical dimerization reaction (EC_2_ reaction) mechanism was proposed during voltametric oxidation [52]. This process produces metastable radicals (B) that consider the initiation material and produce a dication molecule (C), as described in Scheme 2.

## 3. Experimental Work

### 3.1. Chemical Reagents

Chloroauric acid (HAuCl_4_·4H_2_O) (procured from Jinan Future Chemical Co., Ltd., Jinan, China; H_3_PO_4_, NaOH, CH_3_COOH, and K_3_Fe(CN)_6_ (procured from BHD, London, UK); H_3_BO_3_ (from Ntl Nen Tech Ltd., London, UK); KCl (MP Biomedicals LLC, Santa Ana, CA, USA); graphite (2000 Mesh); and paraffin oil (Techno Pharmchem Haryana, Bahadurgarh, India) were used as received. CR was purchased from HIMEDIA Laboratories Pvt. Ltd. (Maharashtra, India). In addition, distilled water was used during the preparation and dilution of the stock solution.

### 3.2. Instrumentation

X-ray diffraction (XRD) was measured using a Bruker D8 Advance with a Cu source, voltage 40 kV, current 40 mA, and a scan speed of 1.5 degrees/min and increment of 0.02 degrees (High-Resolution Ka1 Parallel Beam). Fourier transform infrared (FTIR) spectroscopy was carried out in the range of 4000–500 cm^−1^ using a KBr pellet on a Perkin Elmer (Spectrum 100) FT-IR spectrometer (resolution of 0.5 cm^−1^). The UV–Vis analysis was recorded in the wavelength range 250–800 nm on a Shimadzu UV/1650 spectrophotometer (resolution of less than 2 nm). Transmission electron microscopy (TEM) imaging was performed on an aberration-corrected Titan Cubed 80–300 equipped with a Super-X detector operated at 300 kV using electrodeposited AuNPs on an indium tin oxide (ITO) glass electrode (1 cm × 1 cm with a thickness of 1.1 mm, <10 ohms). Scanning electron microscopy (SEM) was performed on a Helios G4 (Thermo Fisher Scientific, Waltham, MA, USA) equipped with an energy-dispersive X-ray spectrometer (EDX). An Oakton 2700 pH meter was used to adjust the pH of the solution. All electrochemical measurements were performed on an SP-200 Biologic potentiostat/galvanostatic equipped with EC-lab software. The measurements were carried out via a one-compartment three-electrode cell consisting of a CPE (70% graphite and 30% paraffin oil) as the working electrode, Ag/AgCl (3 M KCl) as the reference electrode, and a Pt rod (with 3 mm diameter) as the counter electrode.

The cyclic voltammetry (CV) technique was performed in an applied potential range of −0.2 to 0.8 V in 2 mM K_3_[Fe(CN)_6_] in 0.1 M KCl solution and a potential range of 0.4 to 0.95 V in buffer solution with a scan rate of 50 mV/s vs. Ag/AgCl. Electrochemical impedance spectroscopy (EIS) was measured at 0.235 V in the frequency range of 1.00 Hz–100 kHz. The electrochemical determination of CR was achieved using square-wave voltammetry in the potential range of 0.4 to 1.1 V, with a pulse width (P_w_) of 50 ms, pulse height (P_H_) of 25 mV, and step height (S_H_) of 10 mV.

### 3.3. Preparation of the Modified AuNP/CPE Electrode

The homogenous paste was fabricated using a mixture of 70% graphite and 30% paraffin oil, and the paste was rammed in an electrode hole. The electrode surface was smoothed with gentle polishing by using Sandpaper Silicon Carbide (grade 2000), after which the electrode was polished using filter paper. The prepared electrode was immersed in 2 mM HAuCl_4_·4H_2_O in 0.1 M KCl, and the electrodeposition process was achieved throughout the CV technique in the potential range of 0.2 to 1.0 V at a scan rate of 50 mV/s and different numbers of sweeps.

## 4. Conclusions

In summary, AuNPs were successfully electrodeposited on the CPE via CV technique. The fabricated sensor was used for the sensitive determination of CR dye in 0.1 M ABS at pH 6.5. The AuNP/CPE sensor displayed excellent stability for two weeks, exhibiting high selectivity and reproducibility. Furthermore, the sensor demonstrated a low detection limit of 0.07 μM in the low-concentration range and 0.7 μM in the high-concentration range of CR dye in acetate buffer. Finally, fair recoveries in the range of 88–106% in the different real samples were achieved.

## Figures and Tables

**Table 1:** Previous studies for the detection of CR dye.

Method	Linear Range (µM)	LOD (µM)	Ref. No.
Electro-oxidation at GCE	-----	1.07	[52]
DPV at GO/GCE	0.01–0.2	0.24	[13]
UV-Visible absorption spectra	1.15–345	0.86	[11]
SPMIP-HPLC	-----	0.1 × 10^−3^	[9]
Fluorescent sensor	0.72–71.8 71.8–244	0.05 0.04	[8]
SWV at AuNPs/CPE	1–30 50–200	0.07 0.70	This work

GCE: glassy carbon electrode, DPV: differential pulse voltammetry, GO: graphene oxide, and SPMIP: solid-phase molecular imprinted polymer.

**Table 2:** Recovery of CR dye in real samples.

Sample	C_add_ [μM]	C_found_ [μM]	Recovery [%]
Jelley	30	27.08	90.27
	200	198.12	99.06
Wastewater	30	30.99	103.29
	200	212.39	106.19
Soil	30	28.32	94.41
	200	211.59	105.79
Tap water	30	28.29	94.33
	200	180.29	90.15
Candy	30	26.53	88.44
	200	212.68	106.34

## Data Availability

Not applicable.

## References

[1] El Qada E.N., Allen S.J., Walker G.M. (2008). Adsorption of basic dyes from aqueous solution onto activated carbons. Chem. Eng. J..

[2] Brüschweiler B.J., Merlot C. (2017). Azo dyes in clothing textiles can be cleaved into a series of mutagenic aromatic amines which are not regulated yet. Regul. Toxicol. Pharmacol..

[3] Sarkar S., Banerjee A., Halder U., Biswas R., Bandopadhyay R. (2017). Degradation of Synthetic Azo Dyes of Textile Industry: A Sustainable Approach Using Microbial Enzymes. Water Conserv. Sci. Eng..

[4] Chen X., Deng Q., Lin S., Du C., Zhao S., Hu Y., Yang Z., Lyu Y., Han J. (2017). A new approach for risk assessment of aggregate dermal exposure to banned azo dyes in textiles. Regul. Toxicol. Pharmacol..

[5] Kariyajjanavar P., Jogttappa N., Nayaka Y.A. (2011). Studies on degradation of reactive textile dyes solution by electrochemical method. J. Hazard. Mater..

[6] Zamora M.H., Martínez-Jerónimo F., Cristiani-Urbina E., Cañizares-Villanueva R.O. (2016). Congo red dye affects survival and reproduction in the cladoceran Ceriodaphnia dubia. Effects of direct and dietary exposure. Ecotoxicology.

[7] Hernández-Zamora M., Martínez-Jerónimo F. (2019). Congo red dye diversely affects organisms of different trophic levels: A comparative study with microalgae, cladocerans, and zebrafish embryos. Environ. Sci. Pollut. Res..

[8] Liu L., Mi Z., Wang J., Liu Z., Feng F. (2021). A label-free fluorescent sensor based on yellow-green emissive carbon quantum dots for ultrasensitive detection of congo red and cellular imaging. Microchem. J..

[9] Liu F., Zhang S., Wang G., Zhao J., Guo Z. (2015). A novel bifunctional molecularly imprinted polymer for determination of Congo red in food. RSC Adv..

[10] Morris B. (2003). The components of the Wired Spanning Forest are recurrent. Probab. Theory Relat. Fields.

[11] Sahraei R., Farmany A., Mortazavi S., Noorizadeh H. (2012). Spectrophotometry determination of Congo red in river water samples using nanosilver. Toxicol. Environ. Chem..

[12] Jin L.-N., Qian X.-Y., Wang J.-G., Aslan H., Dong M. (2015). MIL-68 (In) nano-rods for the removal of Congo red dye from aqueous solution. J. Colloid Interface Sci..

[13] Shetti N.P., Malode S.J., Malladi R.S., Nargund S.L., Shukla S.S., Aminabhavi T.M. (2019). Electrochemical detection and degradation of textile dye Congo red at graphene oxide modified electrode. Microchem. J..

[14] Ahmad A., Senapati S., Khan M.I., Kumar R., Sastry M. (2003). Extracellular Biosynthesis of Monodisperse Gold Nanoparticles by a Novel Extremophilic Actinomycete, *Thermomonospora* sp.. Langmuir.

[15] Ahmad A., Senapati S., Khan M.I., Kumar R., Sastry M. (2005). Extra-/Intracellular Biosynthesis of Gold Nanoparticles by an Alkalotolerant Fungus, *Trichothecium* sp.. J. Biomed. Nanotechnol..

[16] Philip D. (2010). Honey mediated green synthesis of silver nanoparticles. Spectrochim. Acta Part A Mol. Biomol. Spectrosc..

[17] Thakkar K.N., Mhatre S.S., Parikh R.Y. (2010). Biological synthesis of metallic nanoparticles. Nanomed. Nanotechnol. Biol. Med..

[18] Ganash A., Almonshi R. (2017). Effects of types and the ratio of poly(o-phenetidine)/TiO_2_ nanocomposite as anticorrosive coating. Polym. Bull..

[19] Ganash A.A., Aljubairy R.A. (2022). Efficient electrochemical detection of hazardous para-nitrophenol based on a carbon paste electrode modified with green synthesized gold/iron oxide nanocomposite. Chem. Pap..

[20] Ganash A.A., Alghamdi R.A. (2021). Fabrication of a novel polyaniline/green-synthesized, silver-nanoparticle-modified carbon paste electrode for electrochemical sensing of lead ions. J. Chin. Chem. Soc..

[21] Haiss W., Thanh N.T.K., Aveyard J., Fernig D.G. (2007). Determination of Size and Concentration of Gold Nanoparticles from UV−Vis Spectra. Anal. Chem..

[22] Huang D., Liao F., Molesa S., Redinger D., Subramanian V. (2003). Plastic-Compatible Low Resistance Printable Gold Nanoparticle Conductors for Flexible Electronics. J. Electrochem. Soc..

[23] Kim H.S., Lee D.Y. (2018). Near-Infrared-Responsive Cancer Photothermal and Photodynamic Therapy Using Gold Nanoparticles. Polymers.

[24] Krishnamurthy S., Esterle A., Sharma N.C., Sahi S.V. (2014). Yucca-derived synthesis of gold nanomaterial and their catalytic potential. Nanoscale Res. Lett..

[25] Sneha K., Sathishkumar M., Kim S., Yun Y.-S. (2010). Counter ions and temperature incorporated tailoring of biogenic gold nanoparticles. Process. Biochem..

[26] León E.R., Rodríguez-Vázquez B.E., Martínez-Higuera A., Rodríguez-Beas C., Larios-Rodríguez E., Navarro R.E., López-Esparza R., Iñiguez-Palomares R.A. (2019). Synthesis of Gold Nanoparticles Using Mimosa tenuiflora Extract, Assessments of Cytotoxicity, Cellular Uptake, and Catalysis. Nanoscale Res. Lett..

[27] Etesami M., Karoonian F.S., Mohamed N. (2011). Electrochemical Deposition of Gold Nanoparticles on Pencil Graphite by Fast Scan Cyclic Voltammetry. J. Chin. Chem. Soc..

[28] Lee K.X., Shameli K., Miyake M., Kuwano N., Khairudin N.B.B.A., Mohamad S.E.B., Yew Y.P. (2016). Green Synthesis of Gold Nanoparticles Using Aqueous Extract of *Garcinia mangostana* Fruit Peels. J. Nanomater..

[29] Zhang Y., Schwartzberg A.M., Xu K., Gu C., Zhang J.Z. (2005). Electrical and thermal conductivities of gold and silver nanoparticles in solutions and films and electrical field enhanced Surface-Enhanced Raman Scattering (SERS). Proceedings of SPIE—The International Society for Optical Engineering.

[30] Adebayo M.A., Adebomi J.I., Abe T.O., Areo F.I. (2020). Removal of aqueous Congo red and malachite green using ackee apple seed–bentonite composite. Colloid Interface Sci. Commun..

[31] Jiang M.-Q., Jin X.-Y., Lu X.-Q., Chen Z.-L. (2010). Adsorption of Pb(II), Cd(II), Ni(II) and Cu(II) onto natural kaolinite clay. Desalination.

[32] Al-Qasmi N., Soomro M.T., Aslam M., Rehman A.U., Ali S., Danish E.Y., Ismail I.M., Hameed A. (2016). The efficacy of the ZnO:α-Fe_2_O_3_ composites modified carbon paste electrode for the sensitive electrochemical detection of loperamide: A detailed investigation. J. Electroanal. Chem..

[33] Vieira S.S., Magriotis Z.M., Santos N.A., Cardoso M.D.G., Saczk A.A. (2011). Macauba palm (*Acrocomia aculeata*) cake from biodiesel processing: An efficient and low cost substrate for the adsorption of dyes. Chem. Eng. J..

[34] Laviron E. (1979). The use of linear potential sweep voltammetry and of a.c. voltammetry for the study of the surface electrochemical reaction of strongly adsorbed systems and of redox modified electrodes. J. Electroanal. Chem. Interfacial Electrochem..

[35] Rhieu S.Y., Reipa V. (2015). Tuning the Size of Gold Nanoparticles with Repetitive Oxidation-reduction Cycles. Am. J. Nanomater..

[36] El-Deab M.S., Ohsaka T. (2002). Hydrodynamic voltammetric studies of the oxygen reduction at gold nanoparticles-electrodeposited gold electrodes. Electrochim. Acta.

[37] Zhao G., Liu G. (2018). Electrochemical Deposition of Gold Nanoparticles on Reduced Graphene Oxide by Fast Scan Cyclic Voltammetry for the Sensitive Determination of As(III). Nanomaterials.

[38] Gunawardena G., Hills G., Montenegro I., Scharifker B. (1982). Electrochemical nucleation. J. Electroanal. Chem. Interfacial Electrochem..

[39] Grujicic D., Pesic B. (2002). Electrodeposition of copper: The nucleation mechanisms. Electrochim. Acta.

[40] Hezard T., Fajerwerg K., Evrard D., Collière V., Behra P., Gros P. (2012). Gold nanoparticles electrodeposited on glassy carbon using cyclic voltammetry: Application to Hg(II) trace analysis. J. Electroanal. Chem..

[41] Finot M., Braybrook G.D., McDermott M.T. (1999). Characterization of electrochemically deposited gold nanocrystals on glassy carbon electrodes. J. Electroanal. Chem..

[42] Daneshgar P., Norouzi P., Ganjali M.R., Dinarvand R., Moosavi-Movahedi A.A. (2009). Determination of Diclofenac on a Dysprosium Nanowire-Modified Carbon Paste Electrode Accomplished in a Flow Injection System by Advanced Filtering. Sensors.

[43] Thanh N.M., Luyen N.D., Toan T.T.T., Phong N.H., Van Hop N. (2019). Voltammetry Determination of Pb(II), Cd(II), and Zn(II) at Bismuth Film Electrode Combined with 8-Hydroxyquinoline as a Complexing Agent. J. Anal. Methods Chem..

[44] Iqbal S., Ahmad S. (2018). Recent development in hybrid conducting polymers: Synthesis, applications and future prospects. J. Ind. Eng. Chem..

[45] Xu Q., Chen L.-L., Lu G.-J., Hu X.-Y., Li H.-B., Ding L., Wang Y. (2010). Electrochemical preparation of poly(bromothymol blue) film and its analytical application. J. Appl. Electrochem..

[46] Gonçalves L.M., Batchelor-McAuley C., Barros A.A., Compton R.G. (2010). Electrochemical Oxidation of Adenine: A Mixed Adsorption and Diffusion Response on an Edge-Plane Pyrolytic Graphite Electrode. J. Phys. Chem. C.

[47] Hasnat M., Ahamad N., Uddin S.N., Mohamed N. (2012). Silver modified platinum surface/H+ conducting Nafion membrane for cathodic reduction of nitrate ions. Appl. Surf. Sci..

[48] Fletcher S. (2008). Tafel slopes from first principles. J. Solid State Electrochem..

[49] Zhang X., Yu S., He W., Uyama H., Xie Q., Zhang L., Yang F. (2014). Electrochemical sensor based on carbon-supported NiCoO_2_ nanoparticles for selective detection of ascorbic acid. Biosens. Bioelectron..

[50] Malode S.J., Shetti N., Nandibewoor S.T. (2012). Voltammetric behavior of theophylline and its determination at multi-wall carbon nanotube paste electrode. Colloids Surf. B Biointerfaces.

[51] Desimoni E., Brunetti B. (2013). Presenting Analytical Performances of Electrochemical Sensors. Some Suggestions. Electroanalysis.

[52] Iwunze M.O. (2010). Electrooxidation of congo red at glassy carbon electrode in aqueous solution. Res. Rev. Electrochem..

[53] Valera D., Sánchez M., Domínguez J.R., Alvarado J., Espinoza-Montero P.J., Carrera P., Bonilla P., Manciati C., González G., Fernández L. (2018). Electrochemical determination of lead in human blood serum and urine by anodic stripping voltammetry using glassy carbon electrodes covered with Ag–Hg and Ag–Bi bimetallic nanoparticles. Anal. Methods.

[54] Tufa L.T., Siraj K., Soreta T.R. (2013). Electrochemical determination of lead using bismuth modified glassy carbon electrode. Russ. J. Electrochem..

[55] Vu H.D., Nguyen L.H., Nguyen T.D., Nguyen H.B., Nguyen T.L., Tran D.L. (2014). Anodic stripping voltammetric determination of Cd2+ and Pb2+ using interpenetrated MWCNT/P1,5-DAN as an enhanced sensing interface. Ionics.

[56] Fichorova R.N., Richardson-Harman N., Alfano M., Belec L., Carbonneil C., Chen S., Cosentino L., Curtis K., Dezzutti C.S., Donoval B. (2008). Biological and Technical Variables Affecting Immunoassay Recovery of Cytokines from Human Serum and Simulated Vaginal Fluid: A Multicenter Study. Anal. Chem..

[57] Ilieva D., Surleva A., Murariu M., Drochioiu G., Abdullah M.M.A.B. (2018). Evaluation of ICP-OES Method for Heavy Metal and Metalloids Determination in Sterile Dump Material. Solid State Phenom..

[58] Bonancêa C.E., Nascimento G.D., de Souza M., Temperini M.L.A., Corio P. (2006). Substrate development for surface-enhanced Raman study of photocatalytic degradation processes: Congo red over silver modified titanium dioxide films. Appl. Catal. B Environ..

[59] Tomkiewicz M., Klein M.P. (1973). Photochemically induced dynamic nuclear polarization in hydrosol suspensions of Congo Red. J. Am. Chem. Soc..

